# Visualization of lipid directed dynamics of perilipin 1 in human primary adipocytes

**DOI:** 10.1038/s41598-017-15059-4

**Published:** 2017-11-08

**Authors:** Jesper S. Hansen, Sofia de Maré, Helena A. Jones, Olga Göransson, Karin Lindkvist-Petersson

**Affiliations:** Department of Experimental Medical Science, Lund University, BMC, 221 84 Lund, Sweden

## Abstract

Perilipin 1 is a lipid droplet coating protein known to regulate lipid metabolism in adipocytes by serving as a physical barrier as well as a recruitment site for lipases to the lipid droplet. Phosphorylation of perilipin 1 by protein kinase A rapidly initiates lipolysis, but the detailed mechanism on how perilipin 1 controls lipolysis is unknown. Here, we identify specific lipid binding properties of perilipin 1 that regulate the dynamics of lipolysis in human primary adipocytes. Cellular imaging combined with biochemical and biophysical analyses demonstrate that perilipin 1 specifically binds to cholesteryl esters, and that their dynamic properties direct segregation of perilipin 1 into topologically distinct micro domains on the lipid droplet. Together, our data points to a simple unifying mechanism that lipid assembly and segregation control lipolysis in human primary adipocytes.

## Introduction

Perilipin 1 (PLIN1) is a key regulator of lipolysis in human adipocytes, where it is abundantly expressed as a phosphoprotein associated with the adipocyte lipid droplet^1^. Human primary adipocytes are spherical monovacuolar cells, typically 50 to 200 μm in diameter, that contain a single centrally located lipid droplet, which takes up to 90% of the cytoplasm, displacing the nucleus to the periphery of the cell (Supplementary Fig. S1). The lipid droplet is stabilized by a surrounding monolayer of phospholipids, which is decorated with adherent proteins, in particular PLIN1. The activity of PLIN1 is regulated in a protein kinase A (PKA)-phosphorylation dependent manner, and its phosphorylation is known to rapidly initiate lipolysis^2^. Despite the central role of perilipins in adipocyte lipid metabolism, its exact mechanism of action is still not fully understood. The prevailing view is, however, that PLIN1 acts dually to both suppress basal lipolysis and augment stimulated lipolysis^1,3^. Several *in vivo* and *in vitro* studies, including perilipin-null mice, have confirmed that reduced levels or absence of perilipin results in increased basal lipolysis^4–8^. Loss of perilipin function results in lean and healthy mice that are resistant to diet-induced obesity, insulin resistance and many related metabolic abnormalities^4,6–8^. Humans carrying PLIN1 mutations present an even stronger phenotype that is markedly different from mice. Aberrant PLIN1 function in humans results in reduced fat mass, partial lipodystrophy, severe dyslipidemia, and insulin-resistant diabetes^9^. The differences between mice and human may be due to species differences in adipose tissue or cells, and emphasize the importance in executing experiments using human adipose tissue. Thus, here we are studying human PLIN1 *ex vivo* and *in vitro* to clarify the molecular mechanisms of PLIN1-regulated lipolysis in humans.

Lipolysis is stimulated by catecholamines that act through β-adrenergic receptor signaling to activate PKA, resulting in a phosphorylation cascade that includes phosphorylation of PLIN1 and hormone sensitive lipase (HSL). Upon phosphorylation, PLIN1 releases the previously associated lipogenic proteins to instead recruit phosphorylated HSL and other lipolytic enzymes to the lipid droplet interface to facilitate lipid mobilization^1^. The basal state predisposition of a pool of non-phosphorylated HSL, at the lipid droplet surface, is thought to initiate lipolysis, while recruitment of phosphorylated HSL by phosphorylated PLIN1 acts to amplify the lipolytic response^10^. These functions of the lipolytic scaffold require a closely associated and dynamic interplay of perilipins with lipid substrates and/or other lipid droplet-associated proteins that remain undetermined^11^. Along these lines, we previously identified the glycerol channel AQP7, as a novel interaction partner of PLIN1 in human primary adipocytes^12^. In addition, confocal microscopy images revealed that PLIN1 forms distinct domains on the lipid droplet surface during insulin stimulation, whereas under lipolytic conditions the distinct protein segregation is abolished resulting in a more homogenous staining pattern^12^. Thus, here we sought to investigate the segregation phenomenon of PLIN1 further. We present evidence for dynamic and hormonally regulated lipid-directed segregation of PLIN1 into topologically distinct micro domains and show that PLIN1 interacts specifically with certain lipids in the cell, such as triacylglycerol (TG), 1,2-dipalmitoyl-*sn*-glycero-3-phosphocholine (DPPC) and cholesteryl esters (CE). The protein-lipid interactions are likely to be crucial for the formation of the PLIN1-rich dynamic micro domains, which in turn may contribute to the regulation of the lipolytic rate in human adipocytes.

## Results

### PLIN1 localizes to domains on the lipid droplet, which are dispersed upon lipolysis

**Figure 1:**
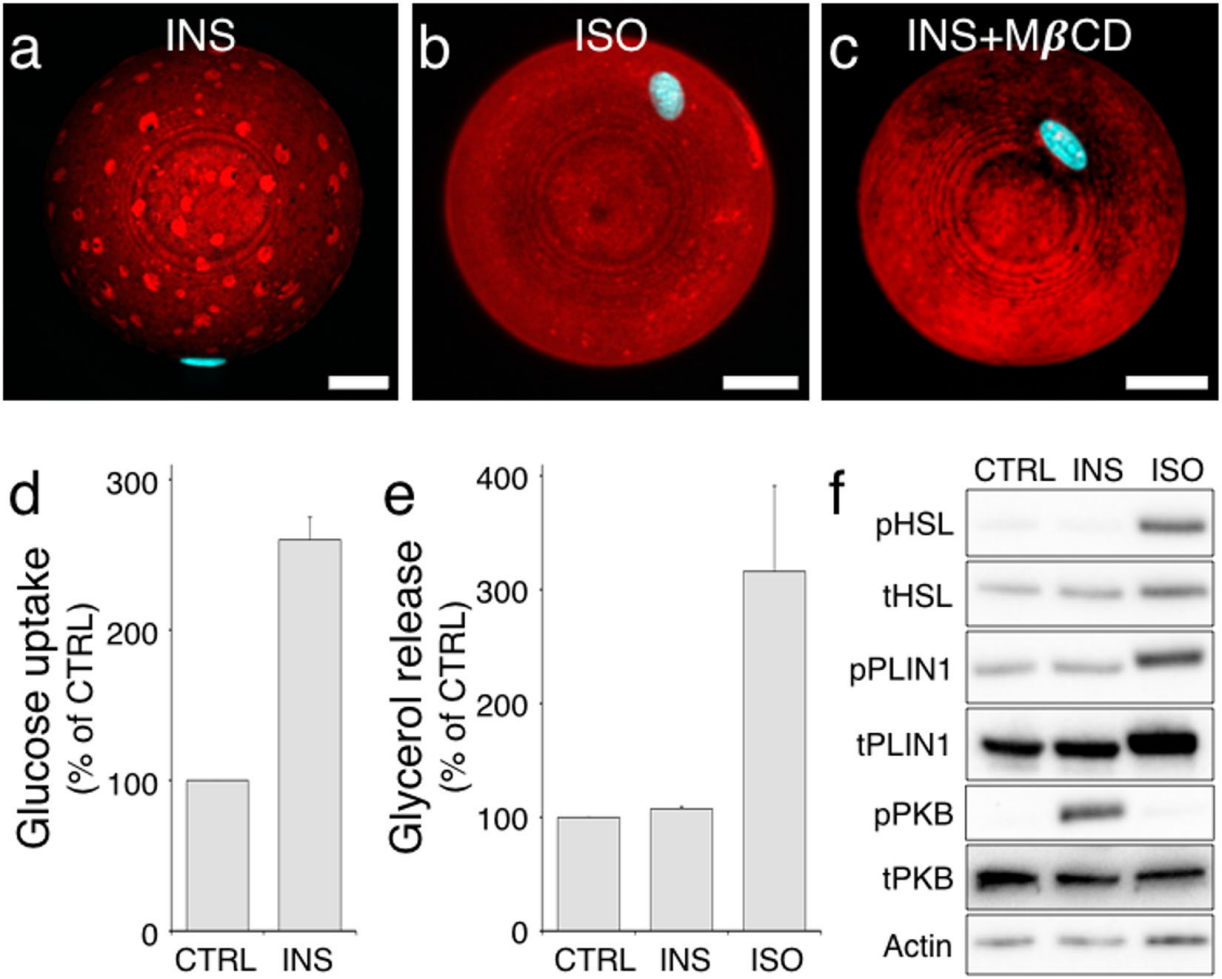
Hormone and MβCD stimulated human primary adipocytes. Standard deviation projections from 3D z-stacks of hormone-stimulated adipocytes immunohistochemically stained for PLIN1. (**a**) Insulin-stimulated (INS). (**b**) Isoprenaline–stimulated (ISO). (**c**) INS-stimulated adipocyte in presence of 10 mM methyl-β-cyclodextrin. PLIN1 staining is shown in red and DAPI nuclei stain is blue. Scale bars are 25 μm. The responsiveness of human primary adipocytes during hormonal treatment was verified by standard glucose uptake and glycerol release assays. (**d**) Glucose uptake during INS stimulation relative to untreated controls (CTRL). (**e**) Glycerol release during INS and ISO treatment relative to CTRL. Results are presented as mean + standard deviation of n = 3 experiments. (**f**) Western blot analysis of lysates from INS and ISO-treated human adipocytes, where p denotes phosphorylated protein and t total protein. The indicated proteins phosphorylation status was assessed using antibodies against the human pHSL S552 (S563 in rat), pPLIN1 S522 (equivalent to murine site S517) and pPKB S473. Also see Supplementary Fig. S2.

To study the details of PLIN1 dynamics in human adipocytes, we applied standard double immunofluorescence staining *ex vivo* in isolated human primary adipocytes. The adipocytes were permeabilized and stained using a primary PLIN1-specific antibody and a fluorescent secondary antibody, and the protein distribution was visualized by confocal laser-scanning microscopy (CLSM). In order to obtain a detailed view of the PLIN1 distribution, we collected stacks of optical sections through the entire adipocyte cell volume. The acquired z-series image stacks were rendered into three-dimensional representations by standard deviation projection to visualize the cellular distribution of PLIN1. In adipocytes under basal conditions (presence of insulin), standard deviation projections from 3D z-stacks show topologically distinct domains enriched in PLIN1 (Fig. 1a), as per our previously published results^12^. Upon stimulation with the β-adrenergic receptor agonist isoprenaline, the PLIN1 domains are abolished and instead a more homogenous PLIN1 stain is observed (Fig. 1b). As perilipins have been shown to sequester to certain lipid droplets^13^, we investigated whether the morphologically distinct domains seen for PLIN1 under lipogenic conditions are dependent on the distribution of different lipids in the monolayer of the lipid droplet, i.e. lipid micro domains. Hence, adipocytes where treated with methyl-β-cyclodextrin (MβCD), a substance frequently used to manipulate the lipid composition of biological membranes by extraction of cholesterol and with concomitant alterations in cellular CE levels^14^. Interestingly, the segregates of PLIN1 were abolished and instead a homogenous stain for PLIN1 was appearing after MβCD treatment (Fig. 1c), similar to the distribution seen upon isoprenaline stimulation (Fig. 1b). These results suggest that lipid micro domain-like structures exist in human primary adipocyte lipid droplets, and that this molecular feature can contribute to the control of PLIN1 distribution. Since MβCD promotes glucose uptake by affecting GLUT4 function^15^, and stimulate lipolysis in a dose-dependent manner in adipocytes^16^, functional assays aiming to draw specific conclusions about the sole consequences of PLIN1 dispersion after MβCD stimulation, may lead to deceptive results. However, to verify our hormone stimulation protocols, we measured the glucose uptake upon insulin stimulation (Fig. 1d) and glycerol release upon isoprenaline stimulation (Fig. 1e) as well as examined the phosphorylation patterns of PLIN1, HSL and PKB after hormone stimulation (Fig. 1f, Supplementary Fig. S2). As expected, the human primary adipocytes take up glucose and release glycerol during insulin and isoprenaline stimulation, respectively. The phosphorylation pattern during isoprenaline stimulation was also in line with previous reports, showing that HSL becomes phosphorylated at the known PKA site S552 (equivalent to S563 in rat) and PLIN1 at S522 (murine site S517)^17,18^. In addition, insulin induced phosphorylation of PKB on the S473 activity controlling site^19^.

### PLIN1 forms micrometer-sized domains on the lipid droplet surface

**Figure 2:**
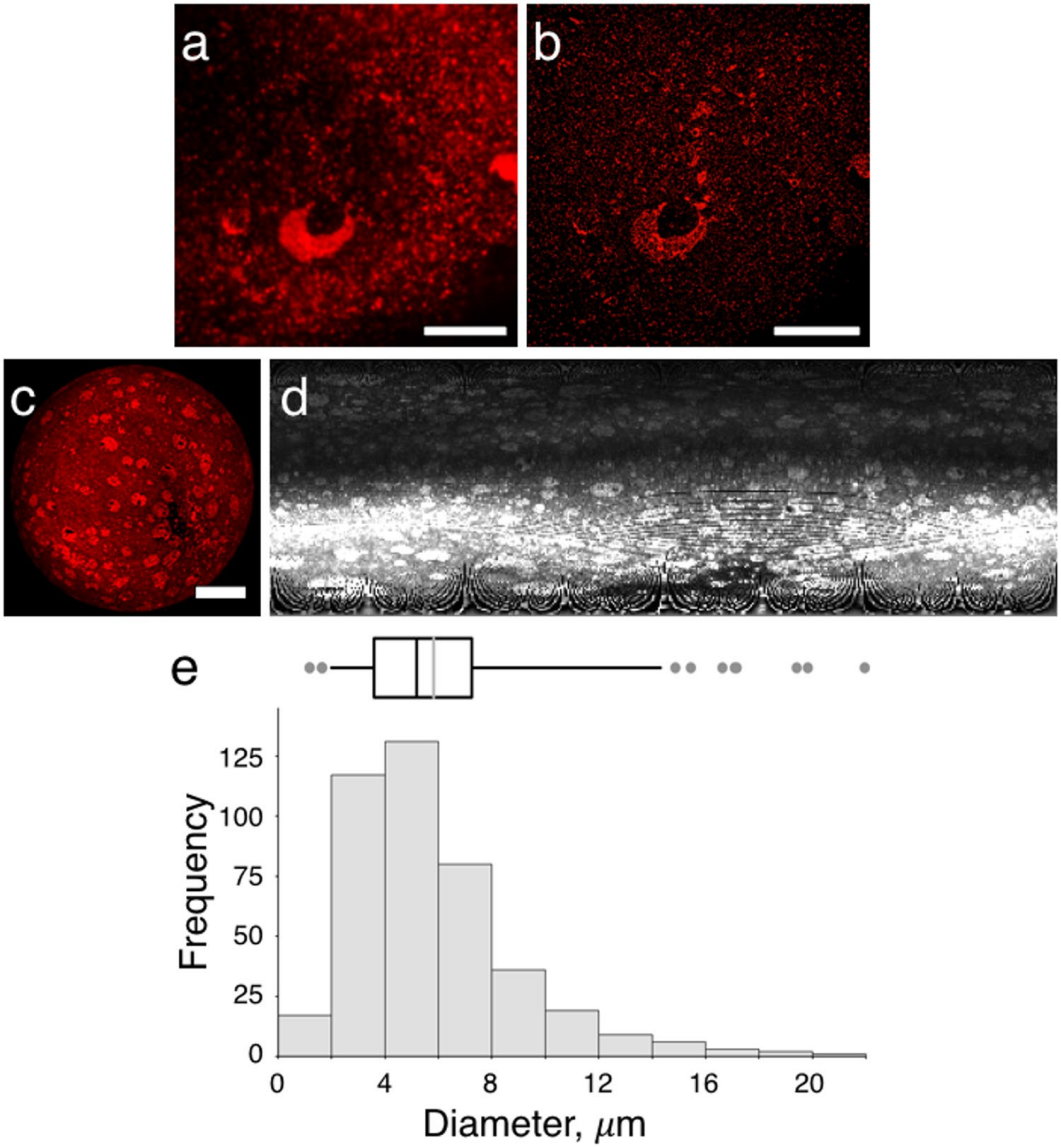
PLIN1 forms spherical micrometer sized patches on the lipid droplet in insulin-stimulated adipocytes. (**a**,**b**) dSTORM super-resolution microscopy, where (**a**) is the 2D TIRF micrograph of INS-stimulated human primary adipocyte stained for PLIN1 (red) prior to dSTORM and (**b**) shows the corresponding dSTORM-generated micrograph. Scale bars are 10 μm. (**c**) CLSM generated 3D projection of INS-stimulated adipocyte immunohistochemically stained for PLIN1 in red. Scale bar is 25 μm. (**d**) Shows the corresponding 2D projection map. (**e**) Size distribution of PLIN1 domains (n = 421) extrapolated from five adipocytes by 2D projection mapping of 3D z-series image stacks. The top inset is the box plot generated from the histogram showing that the PLIN1 micro domains have a median diameter of 5.2 μm (black line) and mean diameter of 5.8 ± 3.1 μm (grey line).

To obtain a more detailed view of the PLIN1-enriched domain morphology during basal conditions, we turned to super-resolution microscopy based on direct stochastical optical reconstruction microscopy (dSTORM)^20–23^. In contrast to CLSM, STORM is a single-molecule localization microscopy technique that enables imaging of specific cellular structures, such as organelles, with nanometer (~20 nm) resolution. The method is based on limiting the number of fluorophores active in each frame of a long series of acquisitions. Multiple rounds of stochastic activation of a photo switchable dye achieve this by brief laser pulses. Here, we used an Alexa Fluor 647-conjugated antibody in blinking mode to achieve dSTORM. Each blinking event during image acquisition corresponds to the specific position of a PLIN1 protein. The centroid of each fluorophore-blinking event is then identified in each frame through rendering, which creates the super-resolution image. We performed dSTORM imaging at a single focal plane at the assumed lipid droplet surface using a TIRF-mode microscope system. The result reveals that the PLIN1-enriched micro domains have a coherent spherical or spherical-like morphology that follows the overall surface curvature of the unilocular lipid droplet, as evidenced by the apparent lack of visible PLIN1 signal outside the assumed droplet surface (Fig. 2a,b). This suggests that the micro domain formation seen for PLIN1 in human primary adipocyte is a distinct lipid dependent segregation, rather than a clustering of protruding lipid droplets, as previously seen in 3T3-L1 cells^13^. We next performed quantitative image analysis by converting 3D fluorescence micrographs into 2D projection maps using Map3-2D software^24^ (Fig. 2c,d, see also Supplementary Fig. S3), and we crudely estimated that the distinct PLIN1 domains have a micrometer size distribution with a median diameter of 5.2 μm (Fig. 2e). These observations confirm that PLIN1 segregates into morphologically distinct micrometer sized domains, and indicate that the micro domain formation of PLIN1 during lipogenic conditions occurs on or near the lipid droplet/lipid monolayer interface.

### PLIN1 does not co-localize with protruding micro lipid droplets in human primary adipocytes

**Figure 3:**
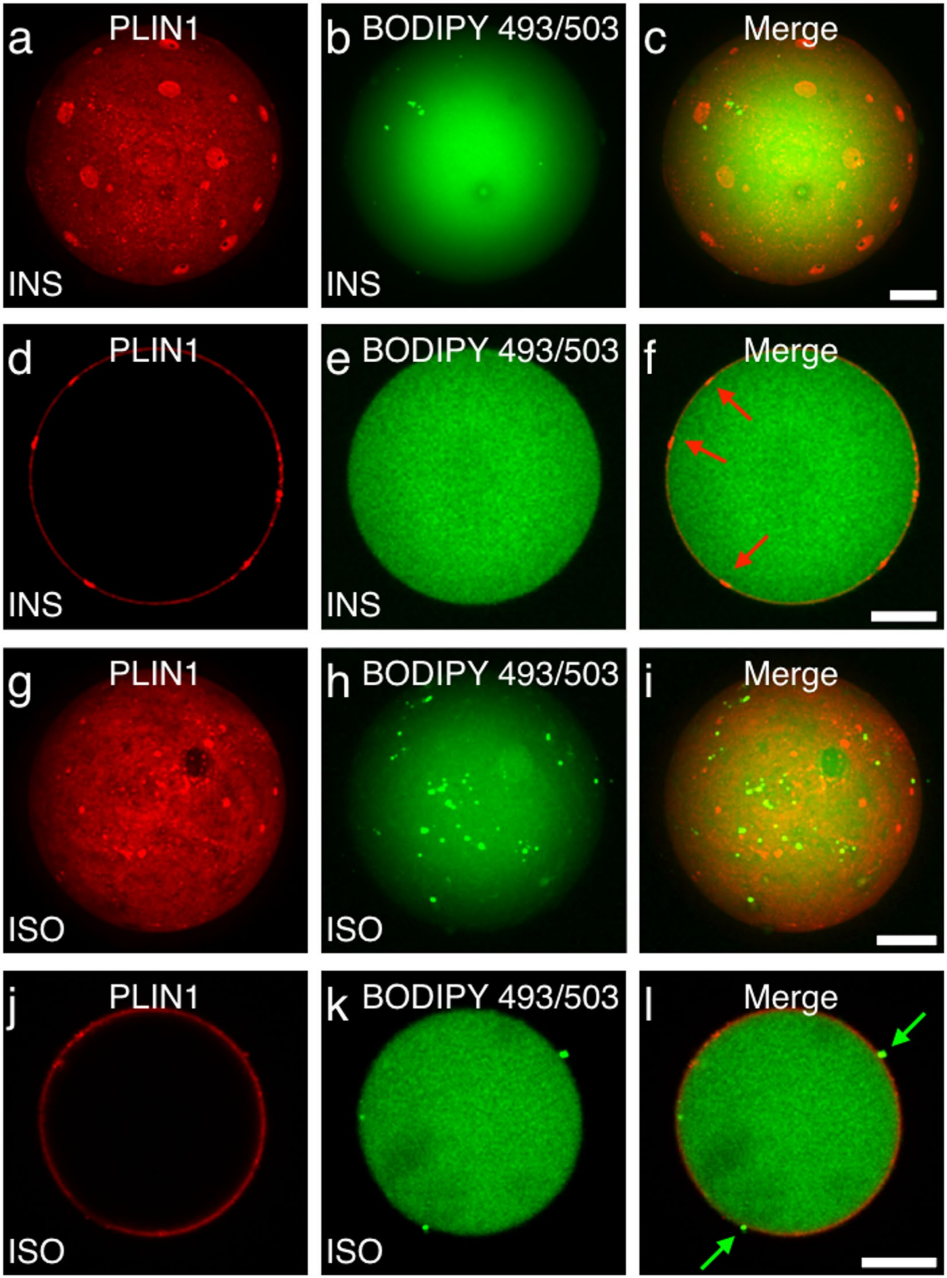
PLIN1 distribution does not coincide with neutral lipid staining. Isolated human primary adipocytes were stimulated with either INS or ISO, and the distribution of PLIN1 and neutral lipids were assessed by microscopy. (**a**–**c**) Standard deviation projection from 3D z-stack of INS-stimulated adipocytes stained for PLIN1 (red) and BODIPY 493/503 (green). (**d**–**f**) Equatorial view of the PLIN1 (red) and BODIPY 493/503 (green) staining pattern in INS-stimulated adipocyte. Red arrows indicate PLIN1 segregation. (**g**,**i**) Standard deviation projection from 3D z-series image stack of ISO-stimulated adipocyte stained for PLIN1 (red) and BODIPY 493/503 (green). (**j**–**l**) Equatorial view of the PLIN1 (red) and BODIPY 493/503 (green) staining pattern in ISO-stimulated adipocyte. Green arrows indicate protruding neutral lipid droplets. Scale bars are 25 μm.

PLIN1 has previously been seen to disperse from the large perinuclear lipid droplet into micro lipid droplets upon lipolysis in the 3T3-L1-mouse cell line as well as in mouse adipose tissue^25,26^. Here, dSTORM imaging indicates that PLIN segregation is a protein-crowding phenomenon at the lipid droplet surface, rather than micro droplet dispersions from the perinuclear lipid droplet, which would have stained as distinct circular shaped rings inside the PLIN1 micro domains in the single focal plane dSTORM image. To clarify this further, we used a neutral lipid stain (BODIPY^TM^ 493/503) to investigate whether it would coincide with the micro domain formations seen with the PLIN1 stain. The BODIPY 493/503 brightly stained the adipocyte interior surrounded by PLIN1, as expected (Fig. 3). Under basal conditions, a few micro lipid droplets are distinctly visible on the large perinuclear lipid droplet, and they seem not to co-localize with the PLIN1 staining pattern (Fig. 3a–c). From the equatorial view of the human primary adipocytes, it is clearly seen that PLIN1 forms a heterogeneously distributed scaffold around the large perinuclear lipid droplet (Fig. 3d–f). The PLIN1 stain forms, as expected, morphological distinct micro domains on the lipid droplet surface, which do not appear to protrude from the lipid droplet (Fig. 3d–f). On the other hand, under lipolytic conditions, many small micro lipid droplets are formed, while the PLIN1-micro domains are abolished (Fig. 3g–i). In an equatorial view, some of the micro lipid droplets are seen to protrude from the large perinuclear lipid droplet (Fig. 3k–l). It is also clear that the PLIN1 staining does not surround those micro droplets, and thus there is no overlap between the PLIN1 stain and the protruding micro lipid droplets formed under these conditions (Fig. 3j–i). Taken together, this data supports the hypothesis that PLIN1 micro domains seen in human adipocytes is a lipid micro domain-like phenomenon.

### Phospholipids create the basis for PLIN1 micro domain formation

**Figure 4:**
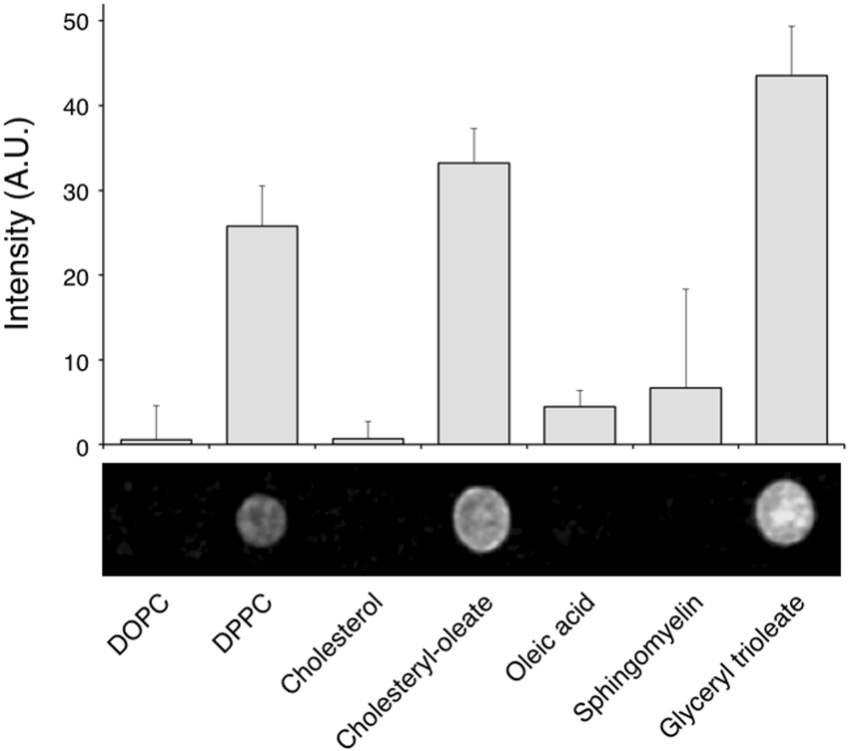
Heterologously expressed PLIN1 has a specific lipid binding profile. A protein lipid overlay assay was applied to investigate the specific interaction of PLIN1 with different lipid droplet constituents. Nitrocellulose membranes pre-spotted with phospholipids, sphingolipid, fatty acid and neutral lipids were incubated with bacterial lysate of PLIN1 recombinant protein stabilized with DM detergent in aqueous solution. The presence of PLIN1 on the dot blots was detected using a PLIN1 specific primary antibody and a fluorescent secondary antibody. The fluorescence intensity on each lipid dot was quantified and plotted for comparison. The inset shows a representative protein:lipid dot-blot experiment. Data are presented as mean + standard deviation of n = 5 experiments.

Lipid micro domains (i.e. lipid rafts) act to facilitate selective protein–protein interactions by excluding or including specific proteins^27,28^. This notion fits well with the suggested central role of PLIN1 acting as recognition site for certain proteins at the lipid droplet/lipid monolayer interface depending on the metabolic state of the adipocyte^29^. Since MβCD ablated the PLIN1 segregation, we aimed to identify what type of lipid species could contribute to the lipogenic segregation of PLIN1. To detect any possible specific interactions between PLIN1 and a lipid component *in vitro*, a lipid overlay assay was applied using heterologously expressed PLIN1 (Fig. 4). This approach enables efficient identification of lipid binding specificities of native three-dimensional protein structures^30^. Hence, PLIN1 was expressed in *E. coli* cells and the PLIN1-containing lysate (PLIN1-lysate) was evaluated for specific lipid binding using the lipid overlay assay approach. Briefly, a subset of lipids specific to adipocytes were spotted onto a nitrocellulose membrane and incubated with PLIN1-lysate^31^. Potential binding of PLIN1 to the lipids was detected using a PLIN1 specific antibody and a fluorescent secondary antibody. A strong PLIN1 signal was detected for the 1,2-dipalmitoyl-*sn*-glycero-3-phosphocholine (DPPC), cholesteryl-oleate and glyceryl trioleate lipid species, but not for the 1,2-dioleoyl-*sn*-glycero-3-phosphocholine (DOPC), cholesterol, oleic acid or sphingomyelin (Fig. 4). Thus, the lipid-binding profile of PLIN1 appears to be very specific, as the protein binds to cholesteryl-oleate, but not cholesterol and to glyceryl trioleate, but not oleic acid. It is also interesting that a strong protein-lipid interaction is observed with the ordered (gel phase) DPPC, whereas the protein has very limited binding affinity with the liquid disordered (fluid phase) DOPC phospholipid. While structural data on PLIN1 is still lacking, functional studies suggest that both PLIN2 and PLIN3 contain independent, non-overlapping, lipid-binding sites involving the 11-mer amphipathic helical motif that is common to all perilipins, including PLIN1^32,33^. PLIN1 additionally has a hydrophobic motif that mediate lipid droplet targeting independently^34^. It is therefore likely that these distinct motifs in PLIN1 are responsible for lipid handling and lipid droplet targeting. For full recognition, both sites may have to be occupied, explaining the distinct lipid-binding profiles for PLIN1 seen in the lipid overlay assay. This suggests that DPPC and neutral lipids are a crucial target for PLIN1, and could contribute to the formation of the PLIN1-rich micro domains observed in the human primary adipocytes under lipogenic conditions.

### Giant vesicles including cholesteryl esters form dynamic micrometer sized micro domains

**Figure 5:**
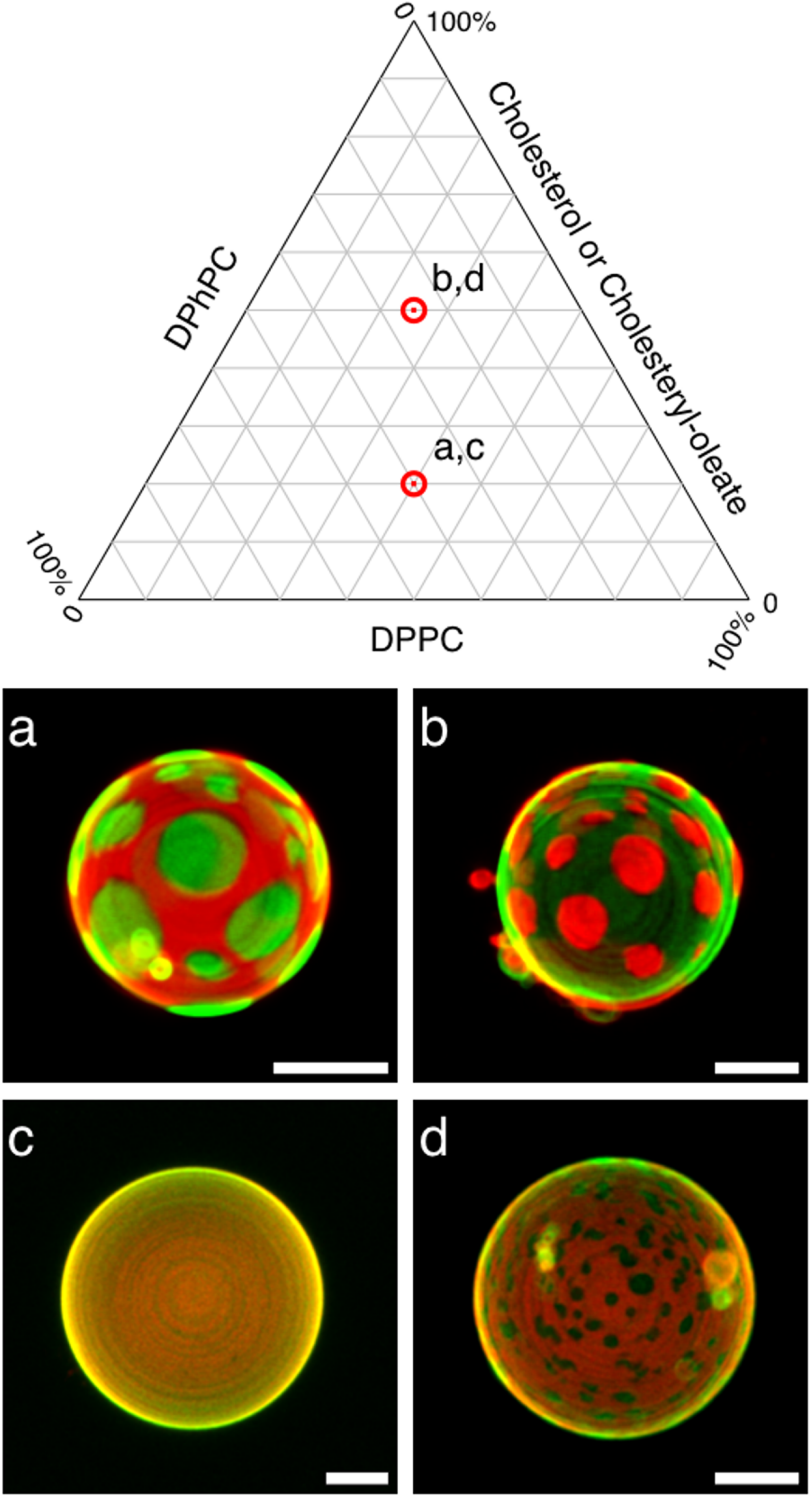
Enrichment of cholesteryl-oleate causes segregation into topologically distinct domains in giant vesicles. Giant vesicles were created with different ternary lipid mixtures of either: DPhPC, DPPC, and Cholesterol; or DPhPC, DPPC and Cholesteryl-oleate. The ternary lipid mixtures were doped with ATTO488 DPPE and Rhodamine DHPE to enable visualization of co-existing ordered (green) and liquid disordered (red) lipid phases, respectively. Shown are standard deviation projections from 3D z-stacks of individual giant vesicles at 30 °C with the ternary lipid mixtures: (**a**) DPhPC:DPPC:Cholesterol, 2:2:1; (**b**) DPhPC:DPPC:Cholesterol, 1:1:2; (**c**) DPhPC:DPPC:Cholesteryl-oleate, 2:2:1; (**d**) DPhPC:DPPC:Cholesteryl-oleate, 1:1:2. Scale bars are 10 μm.

The lipid overlay assay suggests that PLIN1 interacts with TG and CE, in addition to DPPC (Fig. 4). A recent study suggests that neutral lipids (such as TG and CE) are inserted into the phospholipid monolayer to form unique protein recognition sites at the lipid droplet surface^35^. Since PLIN1 is known to form a scaffold that restricts the access of lipases such as HSL to the perinuclear lipid droplet TG content, the specific binding of PLIN1 to TG is not surprising. However, the specific binding to CE is more unconventional. Still, the adipocyte lipid droplet contains high content of CE, which amounts to ~1:2 CE:phospholipids^31^. Nevertheless, the lipid bilayer propensity of CE has, to our knowledge, not previously been investigated. It is well established that ternary lipid mixtures of 1,2-diphytanoyl-*sn*-glycero-3-phosphocholine (DPhPC):DPPC:cholesterol result in coexisting immiscible lipid phases in giant vesicles^36–38^. In the 2:2:1 case, cholesterol-poor regions form the bulk and cholesterol-rich regions are seen concentrated in the smaller domains (Fig. 5a), whereas in the 1:1:2 case, this morphology is inverted by the higher cholesterol content (Fig. 5b). Replacing cholesterol with CE (cholesteryl-oleate) clearly shows that CE has substantial lipid bilayer propensity, and resulted in markedly different miscible/immiscible lipid phase behavior in giant vesicles. With the molar ratio of 2:2:1, giant vesicles formed with apparent complete miscibility and homogenous distribution of lipids is seen throughout the giant vesicle membrane (Fig. 5c). However, in the 1:1:2 case, coexisting immiscible lipid phases are seen, as CE-rich regions concentrated in the smaller domains, resembling the basal state PLIN1 micro domains in human primary adipocytes (Figs 1 and 5d). Thus, in a ternary lipid phase diagram, CE appears to have a fluidizing effect on biological membranes at low to moderate concentrations, while facilitating lipid micro domain formation at high CE-to-phospholipid molar ratios. We believe that this could provide the molecular basis for the regulation and dynamics of PLIN1 in response to hormones.

### Cholesteryl esters co-localize with PLIN1 micro domains in human primary adipocytes

**Figure 6:**
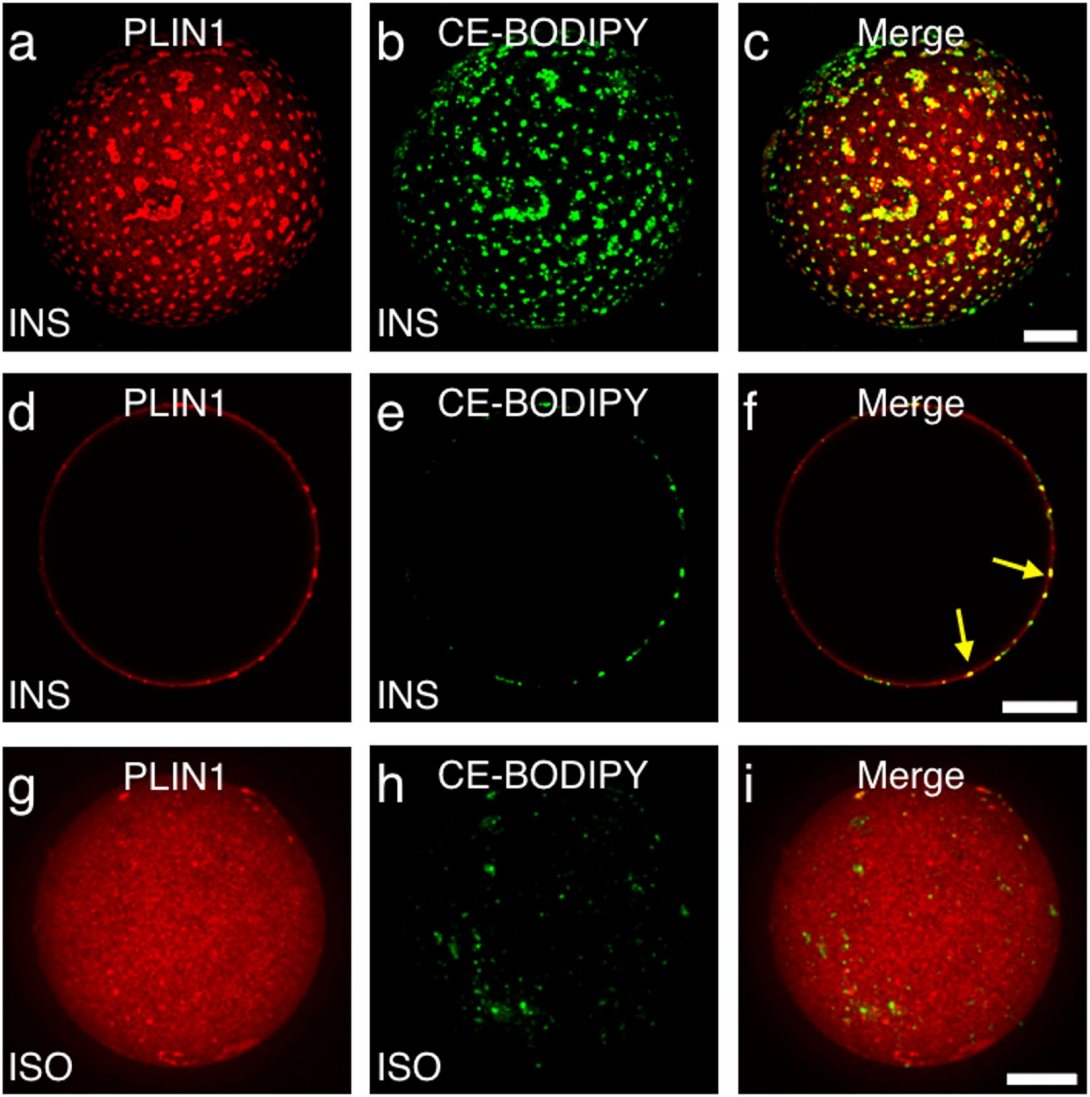
PLIN1 staining co-segregates with CE under lipogenic conditions. Isolated human primary adipocytes were incubated over night in cell medium containing a fluorescent CE tracer, stimulated with either INS or ISO, and subsequently fixed and permeabilized, and immunolabeled for the detection of PLIN1. (**a**–**c**) Standard deviation projection from 3D z-stack of INS-stimulated adipocyte stained for PLIN1 (red) and CE (green). (**d**–**f**) Equatorial view of the PLIN1 and CE staining pattern in INS-stimulated adipocyte. (**g**,**i**) Standard deviation projection from 3D z-stack of ISO-stimulated adipocyte stained for CE and PLIN1. Yellow arrows indicate co-segregation staining of PLIN1 and CE. Scale bars are 25 μm.

To clarify whether CE contributes to the dynamics of the PLIN1 micro domains *ex vivo*, isolated human primary adipocytes were stained for both CE (CholEsteryl BODIPY™ FL C12) and PLIN1 (Fig. 6). Remarkably, the two stains overlap extremely well under basal conditions, clearly suggesting that the specific interaction between CE and PLIN1 shown *in vitro* is likely to take place in human primary adipocytes as well (Fig. 4 and Fig. 6). To investigate whether CE form protruding micro droplets, as have been seen for cultivated cells from rodents^13^, the equatorial view was analyzed. Clearly, the CE itself does not form micro lipid droplets protruding from the unilocular lipid droplet in human primary adipocytes (Fig. 6e). Furthermore, under lipolytic conditions the PLIN1 micro domains disappear, along with the diminished adipocyte CE content, possibly due to HSL dependent hydrolysis (Fig. 6g–i). Thus, based on the previous findings that CE forms dynamic micro domains *in vitro* (Fig. 5), and the fact that PLIN1 binds specifically to CE *in vitro* (Fig. 4), we suggest that CEs have an essential role in the hormonally-regulated dynamics of PLIN1-micro domains, and hence crucial for regulated lipolysis.

### PLIN1 micro domains co-localize with HSL and inhibition of PKA prevents dispersal

**Figure 7:**
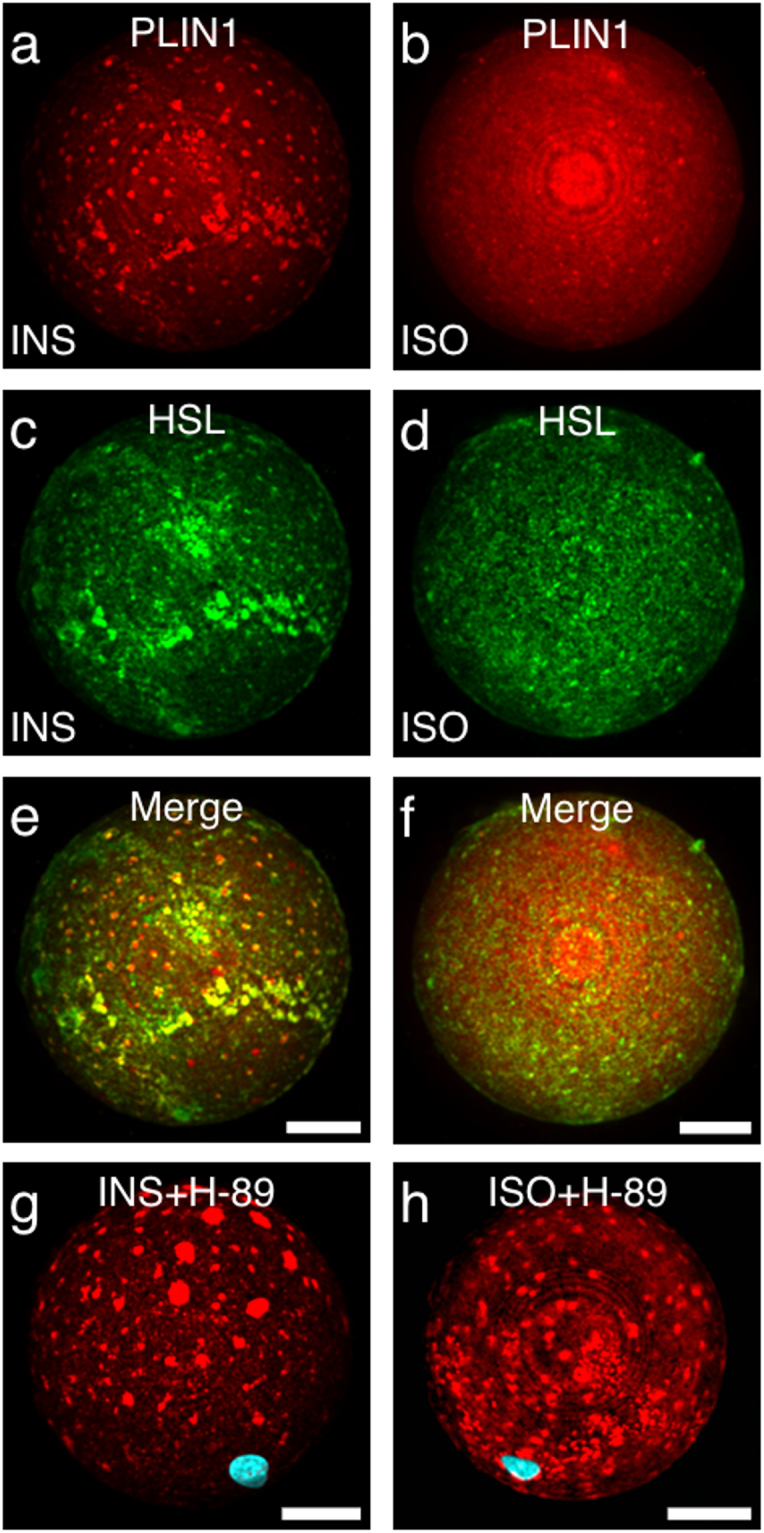
HSL co-localizes with PLIN1 in human primary adipocytes under lipogenic conditions. Following lipogenic or lipolytic stimulation, cells were double immunolabeled for the detection of PLIN1 (red) and HSL (green). Standard deviation projections from 3D z-stacks of individual adipocytes stimulated with either INS (**a**,**c**,**e**) or ISO (**b**,**d**,**f**), respectively, are shown. (**g**,**h**) The effect of PKA on PLIN1 segregation into micro domains was assessed with hormone stimulations in presence of the PKA inhibitor H-89. PLIN1 staining is shown in red and DAPI nuclei stain in blue. Shown are: (**g**) INS-stimulated in presence 25 μM H-89; (**h**) ISO-stimulated in presence of H-89. Scale bars are 25 μm.

The predisposition of non-phosphorylated HSL at the lipid droplet surface is thought to initiate lipolysis, while recruitment of phosphorylated-HSL by phosphorylated-PLIN1 acts to amplify the lipolytic response^10^. Based on the hypothesis that at least part of the lipolytic machinery is preassembled at the lipid droplet surface, including HSL^25,39–43^, we next addressed the distribution between PLIN1 and HSL *ex vivo* in isolated human primary adipocytes. In insulin-stimulated adipocytes, HSL and PLIN1 co-segregate in micro domains (Fig. 7a,c,e), whereas lipolytic stimulation, as mimicked by isoprenaline stimulation, leads a more homogenous coverage on the lipid droplet surface of both proteins (Fig. 7b,d,f). These results support the notion that PLIN1 acts as recognition site for the preassembly of parts of the lipolytic machinery. Still, neither ATGL nor ABDH5/CGI-58 staining coincided with the PLIN1 micro domains (Supplementary Fig. S4), suggesting that they preferably bind to each other or other PLINs such as PLIN5^44–46^. It has previously been speculated that adipocytes may contain a specialized domain devoted to regulating lipolysis and that perilipins may play a key role in organizing the structure and function of this regulatory unit^39^. Our data supports this view. As PKA-mediated phosphorylation is central to the initiation of lipolysis, we next aimed to clarify any possible involvement of PKA in the dispersal of PLIN1 seen in response to lipolytic stimuli. Thus, we repeated the experiment, but incubated the adipocytes with the PKA inhibitor H-89 dihydrochloride and stimulated the cells with either insulin or isoprenaline. As anticipated, the PKA inhibitor did not affect the ability of PLIN1 to concentrate into the distinct spherical micro domains seen during insulin stimulation (Fig. 7g). However, inhibition of the PKA activity resulted in the persistence of the PLIN1 segregation pattern in the presence of isoprenaline (Fig. 7h). This means that PKA-mediated phosphorylation is likely playing a role in the dynamics of hormonally regulated PLIN1 distribution.

## Discussion

It is now well established that adipocytes contribute significantly to the regulation of body energy homoeostasis. Still, analyses of adipocyte cell function have mainly relied on cultured cells, commonly fibroblastic 3T3-L1 cells. There are distinct morphological differences between primary adipocytes and cultured cells, as primary adipocytes have a single large perinuclear lipid droplet, while 3T3-L1 cells have several cytoplasmic lipid droplets. Moreover, the fibroblastic 3T3-L1 pre-adipocytes normally lack endogenous expression of perilipins^47^, meaning that they need to be ectopically expressed. Thus, to study the functional role of the micro domain formations of PLIN1 in human lipolysis, the morphology is crucial, and hence for these studies we selected to apply human primary adipocytes, which abundantly express endogenous PLIN1 and which are naturally highly specialized in lipid handling.

We previously concluded that PLIN1 segregates into micro domains at basal conditions in isolated human primary adipocytes, which disappear upon lipolysis^12^. Since PLIN1 is known to protect the lipid droplet from lipolysis^48^, this intriguing phenomenon could be key to explain the molecular mechanisms of human lipolysis. Thus, in the present study we aimed at resolving the molecular details for the PLIN1 segregation into domains and their dispersal. Our approach consisted of a combination of microscopy studies of isolated human primary adipocytes and biochemical and biophysical analyses of lipid-protein and lipid-lipid interactions. This led us to propose a hypothesis suggesting that DPPC forms the structural basis for micro domain formation, whereas CE plays an essential role for lipolysis, by facilitating the dynamic behavior of PLIN1 micro domains. Moreover, these micro domains proved to be a docking-center for HSL, which is not surprising since HSL is the lipase in adipocytes with CE hydrolysis capability^49^. Taken together, we suggest that; under basal conditions PLIN1 form micro domains by interacting with the gel-phase lipid DPPC and CE, resulting in a fully exposed PLIN1 that can form a physical interaction with HSL^50^. Based on the phase behavior of these lipids in vesicle studies, we believe this provides the molecular basis for the observed PLIN1 enriched micro domains under lipogenic conditions. Upon stimulation of lipolysis by catecholamines, PKA-mediated activation of HSL leads to hydrolysis of CE, resulting in lower levels of CE in the lipid monolayer surrounding the perinuclear lipid droplet, which in turn results in a diffusion of CE micro domains, and consequently a distribution of the PLIN1 micro domains, and thus full lipolysis is initiated. This is supported by the result that upon inhibition of PKA, the micro domains remain persistent, despite lipolytic stimulation (Fig. 7g,h). This fits well with previous reports in 3T3-L1 cells demonstrating that sub-micrometer sized lipid droplets disperse during lipolysis as a consequence of PKA-mediated PLIN1 phosphorylation^51–53^. However, according to our microscopy data, the PLIN1 micro domains do not protrude from the unilocular lipid droplet, and are not stained with neutral lipids, suggesting instead that the PLIN1 micro domains seen in human primary adipocytes is a physical crowding phenomenon at the lipid droplet/lipid monolayer interface. In turn, this creates a docking-center for lipases such as HSL. Early transmission electron microscopy data have shown that PLIN1 distributes heterogeneously on the lipid droplet surface and that the protein protrudes into the lipid triacylglycerol and cholesteryl ester core^54^. As we show here, CE has prominent lipid bilayer propensity and contributes to altered lipid dynamics in lipid bilayers. At low to moderate concentrations, CE diffuse to the liquid disordered bulk phase, while at high concentrations CE-enriched lipid domains are formed. The CE content in human primary adipocytes is delicately regulated by esterification and hydrolysis processes. Under basal conditions, esterification of cholesterol by acyl-CoA cholesterol acyltransferases residing in endoplasmic reticulum produces CE^55^. In human primary adipocytes, the ER membranes wrap around the large perinuclear lipid droplet, facilitating efficient transfer and storage of CE in the lipid droplet^54^. This implicitly also gives rise to a high local CE concentration at the lipid droplet/lipid monolayer interface. As observed in giant vesicles, this can facilitate formation of lipid micro domains. Conversely, during lipolysis activated HSL catalyzes the hydrolysis of CE, thereby lowering the CE content at the lipid droplet/lipid monolayer interface. In turn, this shifts the lipid phase diagram towards complete miscible lipid behavior, alleviating the structural basis for lipid micro domains, and concomitant dispersal of PLIN1. Several lines of evidence support this hypothesis. The phospholipid composition of adipocytes is predominantly phosphatidylcholines, where saturated gel phase DPPC and unsaturated DOPC are the major constituents. Sterols are also present at high levels, comprising overall sterol: phospholipid ratios of 2:1 for cholesterol and 1:2 for CE, respectively^31^. This specific lipid profile is permissible of immiscible lipid membrane behavior^56^, and is able to support organization of lipid micro domains (i.e. lipid rafts) in biological membranes^27,57^. In Langmuir-Blodgett lipid monolayers, pure DPPC forms morphologically distinct spherical domains, which are sensitively modulated in terms of morphology and dynamic properties by even minute fractions of sterols^58^.

Interestingly, another biologically relevant example of lipid–protein monolayers are lung surfactant, necessary to reduce the surface tension in the lung alveoli during respiration^59–61^. Here, surfactant proteins (SP–A to –C) are abundantly expressed as membrane adherent proteins. Lipid binding studies of SP–A to phospholipids have shown that SP-A preferentially binds DPPC, the major lipid component of lung surfactant, but not to other phospholipids^60,61^. The native lung surfactant monolayer also exhibits topologically distinct spherical micro domains, as evidenced by atomic force microscopy^59^. In addition, Bagatolli and co-workers reconstituted surfactant proteins into giant vesicle lipid model membranes of native surfactant lipids, and showed that the proteins segregate into spherical domains, which are redistributed upon MβCD-mediated cholesterol depletion^59^. Thus, the resemblance of the lipid monolayer composition of the adipocyte lipid droplet with lung surfactant is striking and both display micrometer-sized dynamic spherical domains.

In summary, here we propose the hypotheses that lipolysis in human adipocytes is regulated by the dynamics of PLIN1 micro domains. We suggest that the specific control mechanism is lipid segregation, where in particular cholesteryl esters play a crucial role by facilitating the dynamics. Upon hydrolysis by HSL, the local CE content decrease, and thus the lipid micro domain phenomenon terminates, and hence the CE segregation will be abolished along with PLIN1, facilitating the full lipolytic activity seen in human adipocytes.

## Methods

### Ethics

The study was approved by the Regional Ethics Committee, Regionala Etikprövningsnämnden in Lund, (Dnr: 2013/298). All experiments were performed in accordance with relevant guidelines and regulations, and performed after written informed consent obtained from all volunteers. Patients were female without known diabetes (type I or II).

### Immunofluorescence staining of adipocytes

Human primary adipocytes were isolated from abdominal subcutaneous adipose tissue by collagenase digestion^12^. Cells were resuspended in KRB-HEPES containing 25 mM HEPES (pH 7.4), 2 mM glucose, 1% (w/v) BSA and 200 nM adenosine and then stimulated with isoprenaline (1 μM, 10-30 min) or insulin (17.2 nM, 10-30 min) without or with prior treatment with H-89 dihydrochloride hydrate (25 μM, 45 min), or methyl-β-cyclodextrin (10 mM, 60 min). Following, cells were fixed with 4% paraformaldehyde in PBS for 10 min and washed in PBS prior to immunofluorescence staining. The stimulated and fixed human primary adipocytes were permeabilized with 0.1% (w/v) saponin and unspecific antibody binding was blocked with 2% (w/v) fish gelatin in KRB-HEPES (without BSA) for 30 min at 37 °C. Immunofluorescence staining of adipocytes was carried out as described previously using primary antibodies at a dilution of 1:100 and detected using secondary antibodies at a 1:300 dilution^12^. PLIN1 was detected using goat polyclonal to the carboxyterminal end of PLIN1 (Abcam, #ab61682) primary antibody and donkey anti-goat Alexa Fluor 568 (Abcam #ab175704), or donkey anti-goat Alexa Fluor 647 (Abcam #ab150131) secondary antibody. Double immunofluorescence staining of adipocytes was carried out with PLIN1 in combination with HSL, ATGL (Abcam, #ab99532) or CGI-58/ABHD5, respectively. The HSL and ABHD5 antibodies were custom made^62^ and a generous gift from Professor Cecilia Holm (Lund University, Sweden). These antibodies were raised in rabbit and detected using anti-rabbit Alexa Fluor 488 (Abcam #ab150061).

### CE and neutral lipid staining of human primary adipocytes

Isolated adipocytes were incubated overnight in an incubator at 37 °C with 5% CO_2_ in Dulbecco’s Modified Eagle Medium with 25 mM glucose, 3.5% (w/v) BSA, 200 nM N6-(L-2-phenylisopropyl)-adenosine, and Pen/Strep (100 units penicillin and 0.1 mg streptomycin/ml.), supplemented with the fluorescent tracer (1 μM) CholEsteryl 4,4-Difluoro-5,7-Dimethyl-4-Bora-3a,4a-Diaza-s-Indacene-3-Dodecanoate (CholEsteryl BODIPY™ FL C12). The next day, cells were washed 3 times with PBS and then stimulated, fixed and immunofluorescently stained for PLIN1 following the same procedure as described.

Following the described immunofluorescence staining protocol, adipocytes were first stained for PLIN1 and subsequently stained for neutral lipids by incubation with 1 μM BODIPY™ 493/503 (4,4-Difluoro-1,3,5,7,8-Pentamethyl-4-Bora-3a,4a-Diaza-s-Indacene) for 30 min prior to imaging.

### Lipogenesis

Glucose uptake was measured using a fluorometric glucose uptake assay kit (Abcam, USA) following the manufacturer’s instructions.

### Lipolysis

Glycerol release from adipocytes was used as a measure of lipolysis, and was performed using a free glycerol colorimetric/fluorometric assay kit (BioVision Inc., USA) according to the manufacturer’s instructions.

### Stimulation, lysis and Western blot analysis of human adipocytes

Human adipocytes were isolated from abdominal subcutaneous adipose tissue by collagenase digestion. Cells were resuspended in KRB-HEPES containing 25 mM HEPES (pH 7.4), 2 mM glucose, 1% (w/v) BSA and 200 nM adenosine and then stimulated with insulin (10 nM, 10 min) or isoprenaline (30 nM, 30 min). After stimulation, cells were washed in KRB-HEPES (without BSA), and lysed in 50 mM TRIS-HCl (pH 7.5), 0.27 M sucrose, 1 mM EDTA, 1 mM EGTA, 5 mM sodium pyrophosphate, 1 mM sodium orthovanadate, 50 mM sodium fluoride, 1 mM dithiothreitol, 1% (w/v) NP-40 and complete protease inhibitor cocktail (one tablet/50 ml). Lysates were centrifuged at 13,000 × *g* for 15 min (4 °C) and the protein concentration in the supernatant was determined by the Bradford assay. Lysates (5 µg) were then subjected to Western blot analysis, as described previously^63^. The following primary antibodies were used: anti-pHSL S563 (Cell Signaling Technology (CST) #4139, 1:1000), anti-HSL (CST #4107, 1:1000), anti-pPerlipin S522 (Vala Science #4856, 1:1000), anti-Perlipin (CST #9349, 1:5000), anti-pPKB S473 (Thermo Fisher #44621 G, 1:2000), anti-PKB (CST #9272, 1:1000) and anti-β-actin (Sigma #A5441, 1:2000).

### Protein lipid overlay assay

The specific interaction of PLIN1 with phospholipids and sterols was investigated by using a protein lipid overlay assay^30^. Lipids of desired species were dissolved in chloroform (10 mM) and 10 nmol of each lipid were spotted onto nitrocellulose membranes, and dried in a vacuum desiccator for 30 min at room temperature. The membranes were blocked with 0.1% (w/v) BSA and 1% (w/v) fish gelatin in TBS-T (50 mM Tris-HCl (pH 8.0), 150 mM NaCl, and 0.1% (v/v) Tween 20) for 1 h at room temperature. This was followed by 1 h incubation with *E. coli* expressed PLIN1-lysate (A280 = 10) diluted in 20 mM NaPi, pH 8.0, 100 mM NaCl, 5% (v/v) glycerol, 1% w/v n-decyl-β-D-maltopyranoside (DM) supplemented with EDTA-free protease inhibitor cocktail (Roche)^12^. Briefly, a plasmid containing the synthetic human PLIN1 gene was transformed into BL21 (DE3) cells. These cells were cultivated at 37 °C in selective terrific broth medium and protein expression was induced by 1 mM isopropyl β-D-1-thiogalactopyranoside at 18 °C. The cells were lysed by sonication in a buffer containing lysozyme, DNase1, MgSO_4_ and CaCl_2_. Control lysate was produced in parallel in which cells were transformed with the plasmid without PLIN1 gene. The membranes were washed three times in TBS-T, followed by incubation with rabbit anti-PLIN1 monoclonal primary antibody (1:1000) (Cell Signaling, #9349) in TBS-T for 1 h at room temperature with gentle agitation. The membranes were washed three times for 5 min in TBS-T before incubation for 1 h with a secondary 800 nm fluorescent antibody (1:10 000) (LI-COR) in TBS-T at room temperature with gentle agitation. The membranes were washed twice for 5 min in TBS-T to remove excess antibody and the fluorescence signal was recorded using the Odessey Fc gel-scanning system (LI-COR). All images were background corrected using the in-built ImageJ rolling-ball filter, smoothed and the intensity of the fluorescence signal was quantified using ImageJ^64^.

### Giant vesicles

Hydrogel-assisted giant vesicle formation was performed according to established protocols^65^, with the modifications previously described^66^. Briefly, 10 μl of 1% (w/v) molten ultra-low gelling temperature type IX-A agarose (Sigma-Aldrich) were placed onto a plasma-cleaned cover slip (Electro-Technic Inc., USA) and spread evenly by swiping another coverslip across the surface. The cover slips with agarose film were placed in AttoFluor® cell chambers (ThermoFisher Scientific, USA). After gelation of the hydrogel at room temperature, 10 μl of lipid solution (Avanti Polar Lipids and Sigma-Aldrich) dissolved in chloroform (10 mM), with compositions as indicated, were deposited onto the film of agarose and a gentle stream of nitrogen gas ensured immediate evaporation of the solvent. The lipid solutions were doped with 0.2 mol% of ATTO 488 labeled 1,2-dipalmitoyl-*sn*-glycero-3-phosphoethanolamine (ATTO488 DPPE, ATTO-TEC, Germany) and Lissamine™ Rhodamine B labeled 1,2-Dihexadecanoyl-*sn*-Glycero-3-Phosphoethanolamine (Rhodamine DHPE, ThermoFisher Scientific, USA) fluorescent lipid probes to enable visualization of the ordered and liquid disordered lipid phases, respectively^67^. The resultant lipid-hydrogel film was rehydrated with pre-warmed (42 °C) 200 mM sucrose in PBS, pH 7.4. The system was allowed to rehydrate for 10–30 min on a heating block at 42 °C. The formed giant vesicles were harvested by gentle pipetting using wide-bore pipette tips and transferred into warm 200 mM glucose in PBS, pH 7.4 at a 1:5 dilution. The giant vesicles were allowed to settle for 1 h at 42 °C and then transferred to observation chambers pre-equilibrated to 30 °C in a microscope heating stage for viewing.

### Confocal laser-scanning microscopy

Fluorescent micrographs were acquired on a Nikon Confocal A1 + or a Zeiss LSM 510 META confocal laser-scanning microscope. All fluorescence micrographs are background corrected using the in-built ImageJ rolling-ball filter^64^. Standard deviation projections of z-series image stacks were created using standard ImageJ stack tools.

### Direct Stochastic Optical Reconstruction Microscopy Imaging

High-resolution fluorescence microscopy by dSTORM imaging was performed on a TIRF-based Nikon N-STORM Super-Resolution microscope system according to published protocols^20,23^. All imaging was performed using the SR Apochromat TIRF 100 × 1.49 N.A. objective lens. We used the MEA-based imaging buffer as previously described, consisting of 10 mM 2-aminoethanethiol (MEA; Fluka) in 50 mM Tris, pH 8.0, 10 mM NaCl, 10% (w/v) glucose, and 0.5 mg/ml glucose oxidase (Sigma-Aldrich) and 40 μg/ml catalase (Sigma-Aldrich) as the oxygen scavenger system^20^. PLIN1 in human primary adipocytes was detected by immunofluorescence staining using a primary PLIN1-specific primary antibody and the Alexa Fluor® 647 conjugated secondary antibody. This approach can visualize cellular structures with a resolution of approximately 20 nm, which is well below the Abbe diffraction limit^22^. Another advantage with this approach is that the adjustable TIRF angle only penetrates to a depth of only approximately 100 nm into the sample medium, which enables selective visualization of surface regions (reviewed in ref.^20^). The Nikon NIS-Elements software was used for data acquisition, image processing and analysis.

### Statistics

The study includes isolated adipocytes from thirteen patients. Immunolabeled adipocytes were assessed visually by microscopy; typically ≥ 50 cells were viewed per staining condition. Full data collection was performed on a subset. Representative micrographs are presented.

Statistical data is presented as mean + standard deviation of indicated n number of experiments.

## Electronic supplementary material

Supplementary information
