# Supplementary material for: Visualization of lipid directed dynamics of perilipin 1 in human primary adipocytes
Source: Sci Rep. 2017 Nov 8;7:15011. doi: 10.1038/s41598-017-15059-4 (PMC5678101; doi:10.1038/s41598-017-15059-4)
Supplement: Supplementary file 1 — Supplementary information [file 41598_2017_15059_MOESM1_ESM.pdf]

Visualization of lipid directed dynamics of perilipin 1 in human primary adipocytes

Supplementary information

Jesper S. Hansen[1], Sofia de Maré[1], Helena A Jones[1], Olga Göransson[1], Karin Lindkvist-Petersson[1,*]

[1]Department of Experimental Medical Science, Lund University, BMC, 221 84, Lund, Sweden.

[*]Correspondence: karin.lindkvist@med.lu.se

Supplementary figures

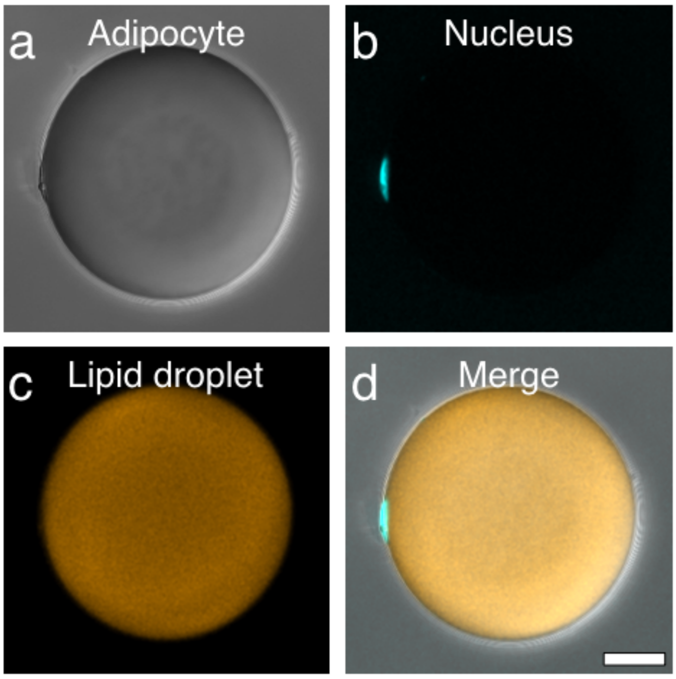

Figure S1 Human primary adipocyte morphology. Cross-sectional 2D brightfield and fluorescence micrographs at a single focal plane showing an unstimulated (i.e. no hormone stimulation) primary adipocyte isolated from human abdominal subcutaneous adipose tissue. (**a**) Brightfield image; (**b**) DAPI nuclei stain; (**c**) Nile Red lipophilic dye; (**d**) Merged micrographs. Scale bar is 25 μm.

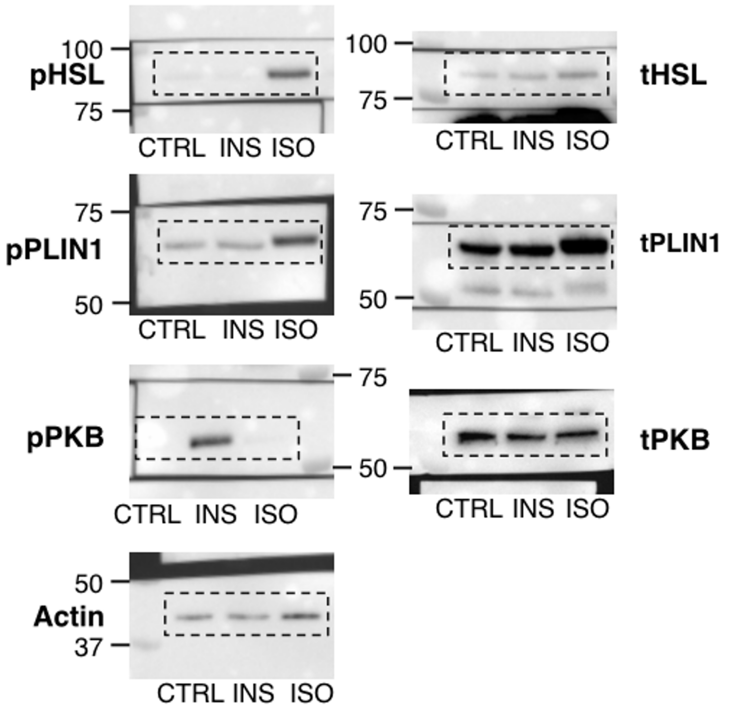

Figure S2 Full length Western blots. Full-length Western blots for main Fig. 1f. The dotted box region is the cropped portion of the blot shown in the main figure.

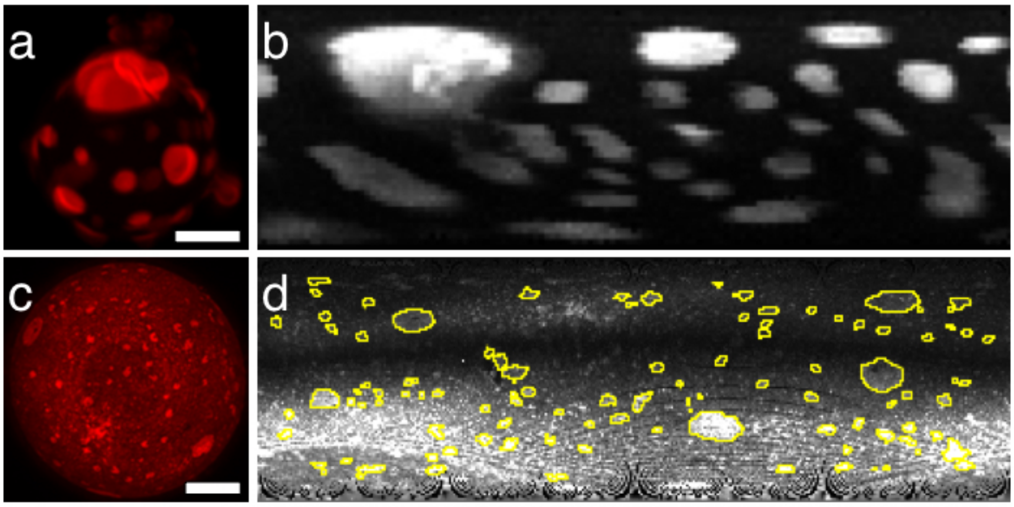

Figure S3 Quantitative 2D projection maps from 3D fluorescence micrographs. 3D z-series image stacks were converted into quantitative 2D projection maps using Map3-2D software. Giant vesicles with a 1:1:2 composition of DPhPC:DPPC:Cholesterol were initially used to evaluate the 2D projection map software. (**a**) Standard deviation projection of a giant vesicle where the Ld phase is shown in red. Scale bar is 10 µm (**b**) Shows the corresponding 2D projection map generated from the raw z-series image stack of the same vesicle. (**c-d**) The same principle exemplified for an INS stimulated adipocyte immunohistochemically stained for PLIN1 (red). Scale bar is 25 µm. Identifiable PLIN1 domains were mapped as region of interest (ROI) from the generated 2D projection maps (**d**). The area of each ROI was then calculated with the inbuilt ROI manager tool in FIJI. Five adipocytes were analyzed in this manner and their PLIN1 domain areas were converted to diameter and histogrammed in main Fig. 2.

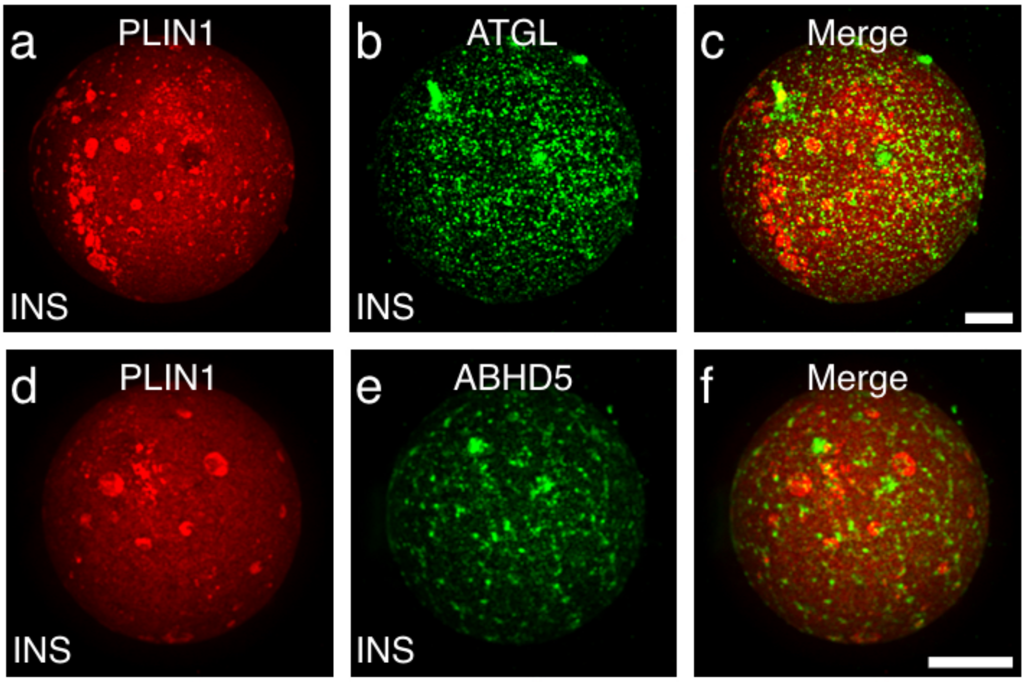

Figure S4 Co-localization of PLIN1 with ATGL and ABHD5 in human primary adipocytes under lipogenic conditions. Following lipogenic stimulation, cells were double immunolabeled for the detection of PLIN1 (red) with ATGL (green) (**a-c**) or with CGI-58/ABHD5 (green) (**d-f**). Standard deviation projections from 3D z-series image stacks of individual adipocytes stimulated with INS are shown. Scale bars are 25 μm.
